# Supplementary material for: Neighbourhood effects on educational attainment. What matters more: Exposure to poverty or exposure to affluence?
Source: PLoS One. 2023 Mar 8;18(3):e0281928. doi: 10.1371/journal.pone.0281928 (PMC9994736; doi:10.1371/journal.pone.0281928)
Supplement: S1 Appendix — (DOCX) [file pone.0281928.s001.docx]

# **Appendix**

**Table 1.** The basic model without spatial variables.

|  | (1) |  |
| --- | --- | --- |
|  | Exposure age 0 – 17 |  |
|  | b | SE |
| Female | 0.325^***^ | (0.008) |
| Household income (in 10k euros, median centered) | 0.158^***^ | (0.003) |
| Western (ref. native Dutch) | 0.071^***^ | (0.018) |
| Non-Western | 0.047^***^ | (0.012) |
| Middle parental education (ref. lower educated) | 0.491^***^ | (0.014) |
| Higher parental education | 1.476^***^ | (0.014) |
| Parental education missing | 0.784^***^ | (0.014) |
| Constant | 15.430^***^ | (0.013) |
| R^2^ | 0.150 |  |

Standard errors in parentheses

^*^ *p* < 0.05, ^**^ *p* < 0.01, ^***^ *p* < 0.001

**Table 2.** VIF values.

|  | (1) |  | (2) |  | (3) |  |
| --- | --- | --- | --- | --- | --- | --- |
|  | Exposure age 0 – 17 |  | Exposure age 0 – 12 |  | Exposure age 13 – 17 |  |
|  | VIF | 1/VIF | VIF | 1/VIF | VIF | 1/VIF |
| Exposure to neighbourhood affluence | 1.56 | 0.640902 | 1.52 | 0.657647 | 1.55 | 0.643576 |
| Exposure to neighbourhood poverty | 1.52 | 0.659378 | 1.47 | 0.678045 | 1.49 | 0.671022 |
| Female | 1.00 | 0.998209 | 1.00 | 0.998211 | 1.00 | 0.998206 |
| Household income (in 10k euros, median centered) | 1.20 | 0.832869 | 1.19 | 0.840395 | 1.20 | 0.833727 |
| Western (ref. native Dutch) | 1.02 | 0.982759 | 1.02 | 0.982980 | 1.02 | 0.983712 |
| Non-Western | 1.33 | 0.751932 | 1.32 | 0.754751 | 1.29 | 0.778015 |
| Middle parental education (ref. lower educated) | 2.64 | 0.379063 | 2.64 | 0.379423 | 2.64 | 0.379347 |
| Higher parental education | 3.07 | 0.326180 | 3.06 | 0.326984 | 3.05 | 0.327461 |
| Parental education missing | 2.80 | 0.357685 | 2.79 | 0.358420 | 2.79 | 0.358343 |
| Urbanicity | 1.25 | 0.797803 | 1.25 | 0.801308 | 1.25 | 0.798701 |
| Equipop distance | 1.19 | 0.840852 | 1.19 | 0.841416 | 1.19 | 0.840197 |
| Mean VIF | 1.69 |  | 1.68 |  | 1.68 |  |

**Table 3a.** Interactions between the exposure to neighbourhood poverty and affluence with parental education.

|  | (1) | | (2) | |  |
| --- | --- | --- | --- | --- | --- |
|  | Interactions with  neighbourhood poverty | | Interactions with  neighbourhood affluence | |  |
| Exposure to neighbourhood poverty (age 0 – 17) | -1.005^***^ | (0.132) | -0.890^***^ | (0.067) |  |
| Exposure to neighbourhood affluence (age 0 – 17) | 2.134^***^ | (0.048) | 2.500^***^ | (0.170) |  |
| *Interaction effects parental education and neighbourhood poverty (ref. is lower educater parents)* | | | | |  |
| Middle edu parents x proportion poor households | -0.792^***^ | (0.163) |  |  |  |
| High edu parents x proportion poor households | 0.941^***^ | (0.164) |  |  |  |
| Parental edu missing x proportion poor households | -0.275 | (0.171) |  |  |  |
| *Interaction effects parental education and neighbourhood affluence (ref. is lower educated parents)* | | | | |  |
| Middle edu parents x proportion affluent households |  |  | 0.219 | (0.187) |  |
| High edu parents x proportion affluent households |  |  | -0.791^***^ | (0.177) |  |
| Parent edu missing x proportion affluent households |  |  | -0.037 | (0.182) |  |
| Urbanicity | 0.328^***^ | (0.011) | 0.322^***^ | (0.011) |  |
| Equipop distance | -0.250^***^ | (0.015) | -0.261^***^ | (0.015) |  |
| Female | 0.309^***^ | (0.008) | 0.403^***^ | (0.025) |  |
| Household income centered | 0.110^***^ | (0.003) | 1.387^***^ | (0.025) |  |
| Western | 0.047^**^ | (0.018) | 0.665^***^ | (0.026) |  |
| Non-Western | 0.147^***^ | (0.013) | 0.403^***^ | (0.025) |  |
| Middle educated parents | 0.536^***^ | (0.028) | 0.403^***^ | (0.025) |  |
| Higher educated parents | 1.166^***^ | (0.027) | 1.387^***^ | (0.025) |  |
| Parental education missing | 0.710^***^ | (0.027) | 0.665^***^ | (0.026) |  |
| Constant | 15.116^***^ | (0.026) | 15.072^***^ | (0.027) |  |
| R^2^ | 0.182 |  | 0.182 |  |  |

Standard errors in parentheses

^*^ *p* < 0.05, ^**^ *p* < 0.01, ^***^ *p* < 0.001
