# Supplementary material for: Neighbourhood effects on educational attainment. What matters more: Exposure to poverty or exposure to affluence?
Source: PLoS One. 2023 Mar 8;18(3):e0281928. doi: 10.1371/journal.pone.0281928 (PMC9994736; doi:10.1371/journal.pone.0281928)
Supplement: Attachment — Submitted filename: Response to Reviewers 2.docx [file pone.0281928.s003.docx]

Dear Dr. Botta,

Thank you very much for the opportunity to revise our manuscript entitled “Neighbourhood effects on educational attainment. What matters more: exposure to poverty or exposure to affluence?” (PONE-D-22-09411R1). And thank you for considering the paper for publication.

We have addressed the reviewers’ comments individually, in the text below. In general, according to the comments of Reviewer 1 we made the text more specific and formal. Following the comments of Reviewer 2, we have added a control variable and we have clarified a number of methodological assumptions and conditions in the text. We feel that the reviewer comments have helped us to further improve the paper and we are grateful for their time.

In one of the comments Reviewer 2 asks us to add a specific reference to our earlier work in the text. In order for the reviewers to still be able to anonymously assess the manuscript, we have replaced this reference in text with “[REDACTED]”. The reference should be:

Troost AA, Janssen HJ, Van Ham M. (2022). Neighbourhood histories and educational attainment: The role of accumulation, duration, timing and sequencing of exposure to poverty. Urban Stud. 2022. DOI: 10.1177/00420980221112351

We hope the revised manuscript meets your expectations.

Best wishes,

Agata Troost, Maarten van Ham and David Manley

**REVIEWER 1**

**COMMENT:**

Reviewer #1: The paper shows that the neighborhood affluence has a stronger effect on educational attainment than neighborhood poverty in the Netherlands.

Even though the paper was improved, there are still inadequate sentences such as "These results highlight the need for more studies on the effects of concentrated affluence, and they can inspire policies focused on the segregation of richer households." - What do you mean "can inspire policies focused on the segregation"? Is segregation something positive in this context? Why?

**RESPONSE:**

We have now rephrased that sentence as: “These results highlight the need for more studies on the effects of concentrated affluence, and they can inspire anti-segregation policies focused on the concentration of rich households” (p. 2).

**COMMENT:**
Several informal expressions such as "Perhaps","In doing so", "This is what we had in mind with the current paper", "according to our assumptions

Excessive use of "" in the paper

**RESPONSE:**

We have made the language more formal and reduced the usage of quotation marks where possible.

**COMMENT:**
Multiple places without references using very strong statements such as "there are theoretical reasons to believe that exposure to affluence may actually be more important as a predictor of educational attainment than exposure to poverty","As already discussed, poverty is often associated with crime and isolation of minority groups."

**RESPONSE:**

For the second cited statement, we have now added references to support it (p. 8):

DeLuca S, Duncan GJ, Keels M, Mendenhall R. The notable and the null: Using mixed methods to understand the diverse impacts of residential mobility programs. In: Neighbourhood effects research: New perspectives. Springer; 2012. p. 195–223.

Sharkey P. Uneasy peace: The great crime decline, the renewal of city life, and the next war on violence. WW Norton & Company; 2018.

The first statement, however, is in the beginning of the conclusion section and summarises our theoretical background section argument, based on multiple studies. We therefore do not think adding one or two references would make sense here, or even referring to all the studies we referenced in theoretical background to make our argument, as the sentence refers to the argument we formulated ourselves. Its function as a summary is clearer while looking at the full sentence (p. 23): “We argued that there are theoretical reasons to believe that exposure to affluence may actually be more important as a predictor of educational attainment than exposure to poverty, because of the crucial influence of interacting with higher educated people on one’s resources, skills and educational aspirations; and, in the Dutch context, because of the lack of extreme concentrated poverty.”

**COMMENT:**
The limitations of the work are not in line with the following statement: "we have painted a fuller picture in which the spatial transmission of poverty is not an isolated problem, but one reinforced by most resources being concentrated somewhere else."

**RESPONSE:**

We have reformulated the sentence as “By studying the effects of living in both affluent and poor environments, we have painted a fuller picture in which urban segregation is not just driven by the sociospatial transmission of deprivation, but also by most resources being concentrated in affluent neighbourhoods” (p. 25).

**COMMENT:**
I am not sure whether it can be also concluded that "Our results support our initial idea that it is often the lack of resources in poor and middle income neighborhoods that is the problem, not the theorized negative effects of poverty itself."

**RESPONSE:**
We have now added more details to make the sentence clearer: “Our results, specifically the effect of spatially concentrated affluence being stronger than that of poverty, support our initial idea that it is often the lack of resources – the cultural and economic capital of richer neighbours - in poor and middle income neighbourhoods that is the problem, not the theorised negative effects of poverty itself” (p. 24).

**COMMENT:**
As the subject of the paper is inequality, I would recommend the authors to tune down the statements (especially the ones that imply "cause and consequence") and use more formal and well accepted jargon.

**RESPONSE:**

We have edited the statements to be more nuanced (as also detailed in the previous comments) and made the language of the paper more formal.

**REVIEWER 2**

**COMMENT:**

Reviewer #2: - Thanks to the authors for making these significant adjustments to the paper. I think the structure and presentation are clearer, and many of comments have been addressed. Kudos!

In general I recommend it for publication, but I would strongly encourage the authors to include these final changes in the submitted version for the reader. I think adding these will help you convince more quantitatively minded readers of your argument.

**RESPONSE:**

Thank you for the kind words and useful comments!

**COMMENT:**
- I could be missing it, but if you do study neighborhood trajectories from the KNN approach in a prior study like you mention in your rebuttal, please reference it when discussing the KNN method or results so that a reader can find that work fairly easily. It is a logical train of thought to want to learn more about the construct you’ve made.

**RESPONSE:**

As the study we mentioned has been published in the meantime, we have added references to it (p. 16, p. 25). We marked the reference in the manuscript as [REDACTED] and sent the actual reference to the editor, as sharing it with the reviewers in possible future rounds of reviews would mean that the review is not double blind anymore.

**COMMENT:**
- I am still a little concerned about the affluent and poverty variables. While not quite a composition, it is close to one as the two variables before being averaged across years are constrained. The correlation is also strongly *negative*, not “not correlated” at -.45. I would just mention this and add an argument why you feel there is no need to transform the variables (e.g. see [https://link.springer.com/chapter/10.1007/978-3-642-36809-7_5](https://urldefense.com/v3/__https:/link.springer.com/chapter/10.1007/978-3-642-36809-7_5__;!!PAKc-5URQlI!7QlJh3z6EcHeVTPuGoAkmDZ5eoDLWB0kSlgi3MDD_KumozLvOaI2OZXCmnsgsBdmDZe69fXdedeMU7GJJT1V$)), or include the VIF for your models to convince the reader that there is not a multicollinearity problem.

**RESPONSE:**

We have now added this explanation, on p. 19: “VIF values were unproblematic, therefore there are no issues with multicollinearity in the models (see Appendix for exact VIF values)”, and also changed “not correlated” to “weakly correlated”.

**COMMENT:**
- I would also include the reasoning you gave me for not including the 'average distance' to different affluent or poor households, mainly because I do not find your answer totally satisfying. You argue that 200 households is a good proxy for social interaction, but the likelihood of interaction with the nearest 200 households for a rural community vs a dense urban one are wildly different in my view. I don't think that controlling for urban density ("urbanity") is enough to capture this. I'd like to see either some text addressing this specifically on e.g. page 16, or some statistics showing me that the urbanization variable highly correlates with a household's avg. distance to poor and affluent households. If it doesn't, I think it would show that there is variation your analysis isn't capturing. Even if that's the case, you can show that and then mention why you don't operationalize it. As it stands it's still a question I have reading the paper.

**RESPONSE:**

Thank you for this comment. We have discussed the issue of the average distance again. Because the correlation between the average distance (measured by Equipop in kilometres necessary to reach the 200 nearest households) and urbanicity (as we decided to rename the variable more accurately) isn’t strong (-0.37 for both high and low income), we decided to add the average distance as a control variable in our models. We believe it helps to control for density of social interaction at a lower level than the urbanicity variable. Adding the variable did not change the significance of any of the other variables except for being of a Western migrant background, but it did influence the strength of the observed effects, making the influence of spatially concentrated affluence stronger than before, and that of poverty a bit weaker. The N of the dataset for analyses changed to 140,338 because of that, but as this indicated that previously some of the bespoke neighbourhood-based variables included squares with missing information for some years, we believe this is an improvement of the study.

**COMMENT:**
- Regarding the “null” model, thanks to the authors for their explanation. However, I would like the explanation you gave me in the review to be present in the text.

**RESPONSE:**

We have now added the explanation to the text on page 19: “The spatial variables contribute around 3% difference in R-squared – from around 15% in models with no spatial variables (for detailed coefficients, see Appendix), through 16% with only the urbanicity control, to 18% with all spatial variables – which is the type of difference that can be expected from similar variables in sociological models. Additionally, including the spatial variables diminishes the effects of other variables in the model, such as family income, which means the spatial variables contribute to the underlying causal structures.”
